# The Pollen Coat Proteome: At the Cutting Edge of Plant Reproduction

**DOI:** 10.3390/proteomes4010005

**Published:** 2016-01-29

**Authors:** Juan David Rejón, François Delalande, Christine Schaeffer-Reiss, Juan de Dios Alché, María Isabel Rodríguez-García, Alain Van Dorsselaer, Antonio Jesús Castro

**Affiliations:** 1 Plant Reproductive Biology Laboratory, Department of Biochemistry, Cell and Molecular Biology of Plants, Estación Experimental del Zaidín, Consejo Superior de Investigaciones Científicas (CSIC), Profesor Albareda 1, 18008 Granada, Spain; juandavid.rejon@hotmail.com (J.D.R.); juandedios.alche@eez.csic.es (J.D.A.); mariaisabel.rodriguez@eez.csic.es (M.I.R.-G.); 2 Bio-Organic Mass Spectrometry Laboratory (LSMBO), IPHC, Université de Strasbourg, 25 rue Becquerel, 67087 Strasbourg, France; delaland@unistra.fr (F.D.); christine.schaeffer@unistra.fr (C.S.-R.); vandors@unistra.fr (A.V.D.); 3 IPHC, Centre National de la Recherche Scientifique (CNRS), UMR7178, 67087 Strasbourg, France

**Keywords:** olive, pollen coat, proteomics, self-incompatibility, tapetum

## Abstract

The tapetum is a single layer of secretory cells which encloses the anther locule and sustains pollen development and maturation. Upon apoptosis, the remnants of the tapetal cells, consisting mostly of lipids and proteins, fill the pits of the sculpted exine to form the bulk of the pollen coat. This extracellular matrix forms an impermeable barrier that protects the male gametophyte from water loss and UV light. It also aids pollen adhesion and hydration and retains small signaling compounds involved in pollen–stigma communication. In this study, we have updated the list of the pollen coat’s protein components and also discussed their functions in the context of sexual reproduction

## 1. Introduction

Sexual reproduction is essential for the propagation of higher plants. From an agronomical point of view, this is a key process as fertilization ensures seed and fruit formation in fruit crop species. Successful pollination and fertilization in higher plants depend on complex interactions between the pollen grain and the pistil tissues, involving adhesion, recognition and pollen tube guidance phenomena [1]. The male gametophyte (pollen), which develops within the anther (the male reproductive organ), comprises a large vegetative cell enclosing a smaller generative cell that often divides to form two sperm cells [2]. Disturbance of anther and/or pollen development could have a detrimental impact on pollen and eventually lead to male sterility [3].

**Figure 1:**
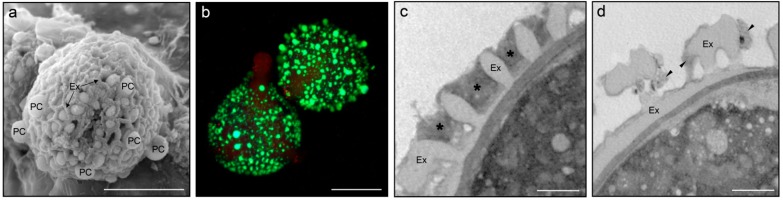
(**a**) Scanning electron microscopy photomicrograph of an olive pollen grain located on the stigmatic surface. The pollen coat (PC) drops fill the cavities of the exine (Ex). (**b**) Olive pollen grains stained with Nile red showing the lipidic nature of the pollen coat (green fluorescence); (**c**,**d**) Transmission electron microscopy photomicrographs of the olive pollen surface before (**c**) and after (**d**) the extraction of the pollen coat (asterisks) with cyclohexane. Arrows in (**d**) denote pollen coat remnants after cyclohexane washing. Bars = 10 µm (**a**,**b**), 1 µm (**c**,**d**).

The male gametophyte cells are surrounded by a unique cell wall that consists of two major strata: an inner pectocellulosic intine, acting as a storage site for hydrolytic enzymes, and an outer exine composed of a complex biopolymer called sporopollenin [2]. The pollen coat, also called “pollenkitt” or “tryphine” [4], is an extracellular matrix derived from the anther tapetum. It is deposited on the outermost surface of the pollen grain following tapetal cell breakdown, filling the spaces and cavities of the highly sculpted exine (Figure 1a). The lipidic nature of the pollen coat is demonstrated by its “stainability” with lypophilic dyes such as Nile red (Figure 1b) and its extractability with organic solvents (Figure 1c,d). The pollen coat is mainly composed of non-polar esters such as sterol esters and saturated acyl groups [5]. Non-polar esters may contribute to maintaining the pollen coat in a semi-solid state, in order to retain proteins and other organic compounds more effectively [6].

The biogenesis of the pollen coat has been intensely studied in the *Brassicaceae* family. In the genus *Brassica*, two tapetal storage organelles, the elaioplasts and the tapetosomes, play a major role in pollen coat formation [5,7]. Elaioplasts are specialized steryl ester-enriched plastids, while tapetosomes are composed of oleosin-coated, triacylglycerol (TAG)-enriched lipid droplets, and cisternae-like vesicles, both of which are assembled in and detached from the endoplasmic reticulum (ER) [8]. After tapetum degradation, elaioplast-derived neutral esters as well as tapetosome-derived flavonoids and alkanes are delivered to the pollen surface to form the pollen coat [8,9,10,11]. Tapetal elaioplasts are also present in other species apart from the *Brassicaceae* family [12,13]. However, species in families other than *Brassicaceae* seem to lack tapetosome-like subcellular structures [14]. Instead, lipid droplets and vesicles appear to be dispersed in the cytoplasm. In the olive tapetum, these organelles were observed to be closely packaged structures resembling the tapetosomes of *Brassicaceae* [15]. The transfer of lipidic material to the pollen surface may occur via capillary action through pressure exerted by the expanding pollen grains against the disintegrating tapetum [16].

In addition to lipids, the pollen coat also contains a number of proteins, most of which are also specifically synthesized in the tapetum layer. Information on how these proteins are delivered to the pollen coat is scarce. In *Brassicaceae*, tapetal oleosins are directly delivered to the pollen coat from tapetosomes [9]. In maize, glucanase, cysteine (Cys) protease and xylanase enzymes are stored in vesicles, vacuoles and the cytosol, respectively, and eventually deposited on the pollen surface following tapetal apoptosis [17]. Some of these proteins undergo further proteolytic processing in the pollen coat [6,9]. Some allergens such as β-expansins are synthetized in the pollen interior and then released to the pollen surface [17]. Other pollen coat proteins are synthesized in both tapetal cells and microspores. Thus, SP11 protein is secreted from tapetal cells to the anther locule and then translocated to the pollen coat [18]. SP11 protein of gametophytic origin may also be secreted to the pollen surface.

In wet-type stigmas, the surface is covered with a viscous extracellular secretion, while dry stigmas, whose surface is coated with a waxy cuticle and a proteinaceous pellicle, lack this secretion (Table S1). The pollen coat may mix with the stigma coating at the point of contact, thus mediating important processes involved in pollination such as pollen adhesion and hydration, pollen–stigma communication and pollen germination. Pollen coat alkanes, or waxes, constitute a relatively impermeable barrier that waterproofs the pollen grain, hence maintaining its water status from the moment of its dispersal until its capture on a compatible stigma. Flavonoids protect the male gametophyte against UV radiation damage during its transport from the anther to a receptive stigmatic surface [11]. Pollen coat lipids and proteins also contribute to pollen adhesion and play a key role in pollen rehydration on dry-type stigmatic surfaces [19,20]. Thus, disruption of pollen coat lipids and proteins in *Arabidopsis* delays or blocks pollen hydration and may result in male sterility [21,22]. The pollen coat also carries the male S-determinant involved in self-incompatibility in the *Brassicaceae* family [18,23]. Last but not least, the adhesive properties of the pollen coat and its content in lipidic volatiles may mediate the entomophilous transmission of pollen grains [4]. In this review, we provide a comprehensive and updated list of pollen coat proteins and also discuss their putative biological functions in plant reproduction.

## 2. The Pollen Coat Proteome

The composition of the pollen coat proteome has been investigated in some plants including species of the *Brassicaceae* family [24,25], cereal crops, such as rice [26] and maize [27], and the olive tree [28]. The pollen coat can be effortlessly extracted by surface washing the intact pollen grains with different organic solvents such as cyclohexane [29], chloroform [27] and diethyl ether [30]. Rinsing times as short as 10 se enable most of the pollen coating material to be removed. Proteins can be purified and solubilized from pollen coat preparations using sonication [29], precipitation with acetone [28], phenol-based extraction methods [31] and detergent-containing buffers [27]. Further analytical separation and identification of proteins can be achieved using either protein microsequencing- or one- or two-dimensional gel-based electrophoretic approaches in combination with mass spectrometry (MS) analysis. Functional validation of pollen coat proteins can be carried out using enzyme and immunocytochemical techniques, *Agrobaterium*-mediated transformation, enzyme and interaction-immunoprecipitation assays of pollen coat-extracted proteins and Western blotting. Table 1 lists the pollen coat proteins identified to date and their putative functions in pollination. A full explanation of each group of proteins is provided below.

**Table 1:** List of pollen coat proteins and their putative biological functions.

Protein Name ^1^	Species ^2^	Putative Function	References
**Acetyl cholinesterases (EC 3.1.1.7)**		Pollen-stigma communication	
Acetyl cholinesterase	*Olea europaea* (EC)		[32]
Cholinesterase	*Vicia faba* (EC)		[33]
**Acylesterases (EC 3.1.1.1)**	*Helianthus annuus* (IGEA)	Pollen tube growths	[34]
**Arabinogalactan proteins (JIM13 epitope)**	*Olea europaea* (IL)	Pollen-stigma adhesion	[35]
**Β-expansins**		Pollen tube growth	
Cyn d 1 *	*Cynodon dactylon* (WB)		[36]
Phl p 1 *	*Phleum pretense* (IL)		[37]
EXPB1a (Ory s 1 *) and OsEXPB13	*Oryza sativa* (MS)		[26]
Expansins B1 and B4	x*Triticosecale* (MS)		[38]
β-expansins-1 and 10 (Zea m 1 *)	*Zea mays* (IL/MS/WB)		[27,39,40,41,42,43]
**Β-glucanases (EC 3.2.1.6)**		Pollen tube growth	
Β-1,3-glucanase	*Zea mays* (SQ/IVEA)		[44]
Ole e 9 *	*Olea europaea* (MS)		[28] (Table S1)
Β-glucanase	*Oryza sativa* (MS)		[26]
Endo-β-1,3-glucanase	x*Triticosecale* (MS)		[38]
**Caleosins**		Pollen-stigma communication	
EF-hand Ca^2+^-binding protein	*Arabidopsis thaliana* (SQ)		[24]
Caleosin	*Brassica napus* (SQ)		[25]
Caleosin	*Olea europaea* (IL/WB)		[15,45]
ABA-induced caleosin	*Zea mays* (MS)		[27]
**Calmodulin-like proteins**		Pollen tube growth	
Bra r 1 *	*Brassica rapa* (IL/WB)		[46]
Serine/threonine kinase	*Olea europaea* (MS)		[28] (Table S1)
**Cysteine proteases (EC 3.4.22)**		Tapetum PCD/Pollen tube growth	
CEP1	*Arabidopsis thaliana* (IL)		[47]
BGP-CP *	*Cynodon dactylon* (MS/WB)		[36]
Phl p CP *	*Phleum pretense* (WB)		[36]
Sor h CP *	*Sorghum halepense* (WB)		[36]
Cysteine protease	*Zea Mays* (IL/MS/SQ/WB)		[27,48]
**GDSL esterases/lipases (EC 3.1.1.-)**		Pollen rehydration/Pollen tube growth	
EXL4 and EXL6 lipases	*Arabidopsis thaliana* (SQ/WB)		[24,49]
GDSL esterase/lipase	*Olea europaea* (MS)		[28] (Table S1)
**Lipases (EC 3.1.1.3)**	*Helianthus annuus* (EC/IVEA)	Unknown	[34]
**Ole e 1 allergen family**		Pollen tube growth	
Ole e 1 *	*Olea europaea* (IL)		[50,51] (Table S1)
Ole e 1-like *	x*Triticosecale* (MS)		[38]
**Pectinesterases (EC 3.1.1.11)**		Pollen tube growth	
Pectin esterase	*Brassica napus* (SQ)		[25]
Ole e 11 *	*Olea europaea* (MS)		[28] (Table S1)
**Pectate lyases (EC 4.2.2.2)**		Pollen tube growth	
Cry j 1 *	*Cryptomeria japonica* (IL)		[52,53]
Cup a 1 *	*Cupressus arizonica* (IL)		[54,55]
Cry j 1-like *	*Cupressus sempervirens* (IL)		[55]
Phl p 4 *	*Phleum pretense* (IL/MS/WB)	Unknown	[36,56]
**Polygalaturonases (EC 3.2.1.15)**		Pollen tube growth	
Polygalacturonase	*Brassica napus* (IL)		[57]
Polygalacturonase	*Olea europaea* (MS)		[28] (Table S1)
Polygalacturonase	x*Triticosecale* (MS)		[38]
Exopolygalacturonase (Zea m 13 *)	*Zea mays* (MS)		[27,44]
**Pollen coat protein, class A (PCP-A)**		Self-incompatibility/Pollen rehydration/Pollen adhesion	
PCP7-like	*Brassica napus* (IVIA)		[58]
PCP7/PCP-A1	*Brassica oleracea* (IVIA)		[29,59,60]
PCP1	*Brassica oleracea* (SQ)		[61]
BcPCP-A1	*Brassica rapa* (IVIA)		[20]
SLR1-BP1 and SLR1-BP2	*Brassica rapa* (IVIA/MS)		[20]
SP11/SCR (male *S*-determinant)	*Brassica rapa* (IL/IVIA)		[18,23,62,63,64]
**Profilins**		Unknown	
Ole e 2 *	*Olea europaea* (IL/MS)		[65]
Profilin/Ory s 12 *	*Oryza sativa* (MS)		[26]
Profilin *	x*Triticosecale* (MS)		[38]
Profilin/Zea m 12 *	*Zea mays* (MS/SQ)		[27,44]
**Receptor-like protein kinases**		Unknown	
Kinase	*Arabidopsis thaliana* (SQ)		[24]
Protein kinase	*Brassica napus* (SQ)		[25]
**Subtilisin-like Ser proteases (EC 3.4.21.-)**		Unknown	
Subtilisin-like Ser protease	*Oryza sativa* (MS)		[26]
Putative subtilase	*Zea mays* (MS)		[27]
**Tapetal oleosins (T-oleosins)**		Pollen rehydration/Tapetosome formation/Pollen dehydration tolerance	
GRP17	*Arabidopsis thaliana* (MU/PAGE/WB)		[22]
GRP14 & GRP16−19	*Arabidopsis thaliana* (PAGE/SQ)		[24]
T3, T5 & T6 oleosins	*Arabidopsis thaliana* (AMT/MU)		[14]
BnOlnB;4	*Arabidopsis thaliana* (AMT/IL/WB)		[66]
BnOlnB;4	*Brassica carinata* (AMT/IL/WB)		[67]
BnOlnB;3, BnOlnB;4 & BnOlnB;6	*Brassica napus* (PAGE/SQ)		[68]
BnOlnB1−6 & 11/Pollenins 1−6 & 11	*Brassica napus* (IL/SQ/WB)		[6,25]
BnOlnB;3 & BnOlnB;4	*Brassica napus* (IL/WB)		[8,9]
39-kDa oleosin fragment	*Brassica oleracea* (SQ/WB)		[9]
BOPC3, BOPC4 & BOPC5	*Brassica oleracea* (IS/PAGE/WB)		[69]
37-kDa oleosin fragment	*Brassica rapa* (SQ/WB)		[8,9]
**Xylanases (EC 3.2.1.8)**		Pollen tube growth	
EXY *	*Cynodon dactylon* (MS/WB)		[36]
30-kDa endoxylanase *	*Phleum pretense* (MS/WB)		[36]
1,4-β-xylanase	*Oryza sativa* (MS/WB)		[26]
Endoxylanase	*Zea Mays* (MS/SQ/WB/IVEA)		[27,30,70,71]

^1^ Allergenic proteins are marked with an asterisk; ^2^ The methods used to study the pollen coat protein are indicated in parentheses. **AMT**, *Agrobaterium*-mediated transformation; EC, enzyme cytochemistry; IGEA, *in gel* (polyacrylamide) enzyme assay of pollen coat-extracted proteins; IL, immunolocalization; IS, immunoscreening of a cDNA expression library; IVEA, *in vitro* enzyme assay of pollen coat-extracted proteins; IVIA, *in vitro* and/or *in vivo* interaction assays; MS, mass spectrometry identification; MU, mutant analysis; PAGE, SDS-PAGE; SQ, protein micro-sequencing; WB, Western blotting.

### 2.1. The Pollen Coat Proteome of Brassicaceae

In the *Brassicaceae*, the pollen coat of the model plant *Arabidopsis thaliana* and several species of the genus *Brassica* was subjected to proteomic scrutiny (Table 1). In this family, tapetal cells synthesize a number of specific oleosin-like proteins (T-oleosins), previously called glycine-rich proteins [24] and oleo-pollenins [25], which are the most abundant proteinaceous components of the pollen coat (Table S2). All T-oleosins comprise a conserved hydrophobic lipid-binding hairpin motif of ~70 nonpolar amino acid residues (AAs), which resembles that of seed and pollen oil-body-associated counterparts flanked by hydrophilic/amphipathic N- and C-terminal regions. The N-terminus is highly variable in length (6–68 AAs) and amino acid composition [68]. The C-terminus is also highly variable in length (28–1000 AAs) and contains repeating motifs [lysine (Lys)/glycine (Gly) and Gly-rich domains and proline (Pro)/alanine (Ala) repeats] that allow these proteins to evolve rapidly [72,73]. T-oleosins are targeted to oil bodies in tapetosomes, stabilizing large cytoplasmic lipid bodies [6,8]. The oleosin-like domain may represent a novel cleavable site that is removed by proteolysis, releasing the C-terminal mature protein, previously called pollenin [25]. Proteolytic activity is likely to occur after oleosin relocation to the pollen surface in order to ensure that only specific T-oleosins persist in the pollen coat.

The T-oleosin cluster has been found in several species of the genus *Arabidopsis*, such as *A. thaliana*, *A. lyrata* and *A. arenosa*, as well as other closely-related *Brassicaceae* species including the genera *Brassica*, *Boechera*, *Capsella*, *Olimarabidopsis* and *Sisimbryum* [72,73]. The genome of *Brassica napus* contains 12 anther-specific oleosin genes (BnOlnB;1–12), six of which (BnOlnB;1–4 and BnOlnB;6–7) are highly expressed in the tapetum from either the early or late uninucleate microspore stages to the bicellular pollen stage [68,74]. At the protein level, four of the 11 most abundant polypeptides (~32–40 kDa) from pollen coat preparations matched with BnOlnB;3-4 oleosins. Another group of five polypeptides with smaller sizes (~6–17 kDa) was identified as BnOlnB;1, BnOlnB;2, BnOlnB;5, BnOlnB;6 and BnOlnB;11 oleosins [6]. The two major T-oleosins are synthesized in tapetosomes as larger precursors of ~48 and ~45 kDa at the early and late microspore developmental stages [6,9,11]. These precursors undergo a proteolytic cleavage to smaller fragments of ~37 and ~35 kDa, respectively [6,8,9]. The oleosin-like domains are mostly degraded although, at least in the case of BnOlnB;1 and BnOlnB;5, they are still detectable on the pollen coat albeit at very low levels [6,25]. A similar T-oleosin cluster is present in the genome of *B. oleracea*, with six genes (BoBlnB;1−6) that are orthologues of AtT1−5 and AtT7 genes, respectively [14]. Expression begins at the uninucleate microspore stage, peaks at the bicellular pollen stage and decreases after the second mitosis [69]. Five polypeptides, with molecular weights of between 34 and 42 kDa, were detected in extracts from the pollen coat of *B. oleracea* [69]. The genome of *B. campestris* (synonym: *B. rapa* ssp. *oleifera*) contains five anther-specific oleosin genes (BrOlnB;1−5), one of which encodes a polypeptide of 45 kDa that is converted to a 37-kDa fragment in the pollen coat [8,9]. A recent study has shown that T-oleosin paralogs found in the *Brassicaceae* family may have been retained in the course of evolution in a lineage-specific manner due to their additive benefit of pollen vigor [14].

The *A. thaliana* genome contains up to nine T-oleosin paralogs encoding proteins with molecular weights of between 10.7 and 53.2 kDa, eight of which are grouped in tandem in a 30-Kb locus on chromosome 5 [72,73]. The nine gene paralogs were found to be specifically transcribed in the tapetal cells with a peak of expression at the vacuolated microspore stage [14,75]. In the *Arabidopsis* pollen coat, the 17.4-kDa T1 and 53.2-kDa T3 oleosins are the most abundant protein constituents, followed by T4, T2, T6 and T5 in that order [22,24]. A recent study has shown that *Arabidopsis* T3 and T5 oleosins are also proteolytically cleaved, generating the corresponding C-terminal fragments [66].

A second group of well-characterized *Brassica* pollen coat-borne proteins comprises a number of basic Cys-rich small proteins of ≤10 kDa, which are grouped in class A of the pollen coat protein (PCP) family [59]. The genome of *B. oleracea* is composed of between 30 and 40 PCP genes. The first protein of this family to be identified was a 7-kDa peptide from the pollen coat of *B. oleracea* called PCP^7^, subsequently renamed PCP-A1 [29,59]. PCP-A1 is encoded by a single gene that is transcribed specifically in the cytoplasm of the maturing pollen, with a peak of expression at the tricellular pollen stage. The nascent polypeptide chain contains 81 AAs, including a cleavable 26-AA signal peptide that suggests its secretion via ER/Golgi. The mature polypeptide is predominantly hydrophylic and is estimated to have a molecular weight of ~6.4 kDa and a pI of 9.26 [59]. A second PCP gene was cloned in *B. oleracea* and named PCP1, which encodes a basic polypeptide of 83 AAs in length, including a 25-AA signal peptide [61]. A PCP-A1-like protein of ~7 kDa was also identified in *B. napus* [58]. In *B. campestris*, two PCP-A proteins, called S locus-related glycoprotein 1-binding protein 1 (SLR1-BP1) and 2 (SLR1-BP2), were cloned and characterized [20]. Both proteins are almost identical, differing only in a P31 that is hydroxylated in SLR1-BP1 but not in SLR2-BP2. The mature form of all PCP-A proteins contains eight highly conserved Cys residues but is extremely divergent at the intervening sequences [59].

The PCP family also contains the male determinant involved in the self-incompatibility response in the *Brassicacea*. The S-locus encoded male determinant was identified and characterized in the late 1990’s by two independent research groups led by Prof. June Nasrallah at Cornell University in New York (USA) and Prof. Akira Isogai at the Nara Institute of Science in Ikoma (Nara, Japan), respectively. Thus, sequence analysis of the S locus glycoprotein (SLG)/S locus receptor kinase (SRK) chromosomal segment of the *S_8_* and *S_9_* haplotypes of *B. campestris* resulted in the identification of two identical S-locus encoded genes, called S-locus Cys-rich protein (SCR) [23] and S-locus protein 11 (SP11) [62], respectively, as potential candidates encoding the pollen S determinant. Further experiments demonstrated that the *SCR/SP11* gene is linked to the S-locus and co-segregates with the *SLG/SRK* gene pair in an S haplotype-specific manner, thus suggesting that *SCR/SP11* and *SLG/SRK* genes have co-evolved [18,23]. Although the alleles of the *SCR/SP11* gene are all located between *SLG* and *SRK,* their relative distances from them and their orientation diverge [18]. SCR/SP11 gene expression, which starts at the early uninucleate microspore stage and ends just before flower anthesis, takes place in both the tapetum and developing microspores [18,23]. SCR/SP11 genes from different *S* haplotypes encode small (~8.0–9.0 kDa), basic and highly polymorphic proteins. This polymorphism is consistent with the function of the *S* male determinant in mediating specificity in the self-incompatibility response. All SCR/SP11 mature polypeptides contain eight conserved cysteines, a hallmark of the PCP family, but differ in their positions with respect to those of PCP-A1, thus suggesting that SCR/SP11 belongs to a novel class of the PCP family. Immunostaining experiments have confirmed that SCR/SP11 proteins show both gametophytic and sporophytic expression (Table S2), which is regulated by different cis-regulatory elements [63,64]. SCR/SP11 protein seems to be secreted from the tapetum to the anther locule and further translocated to the pollen surface, mainly the exine and the pollen coat, hence explaining the sporophytic nature of *Brassica* self-incompatibility [64].

The *A. thaliana* pollen coat proteome also includes two proline-rich extracellular lipases (EXL4 and EXL6), two putative receptor kinases (~31–32 kDa) with extracellular domains and a putative caleosin [24]. Lipases are grouped in a genomic cluster, which contains six paralogs and is located on chromosome 1 [24]. EXL4 and EXL6 are members of the GDSL lipase/esterase family. These proteins share little homology with most lipases and possess a highly conserved catalytic tetrad composed of residues serine (Ser)-Gly-asparagine (Asn)-histidine (His) [76]. The depletion of most of these components in pollen coat-defective *cer* (*eceriferum*) mutants confirms their extracellular nature [21]. On the other hand, the pollen coat of *B. napus* also contains a ~28-kDa caleosin similar to that found in *Arabidopsis*, a protein kinase of 24 kDa that is smaller than the two identified in *Arabidopsis*, and a ~20-kDa pectinase enzyme [25].

### 2.2. The Pollen Coat Proteome of Grasses

The composition of the pollen coat proteome has also been investigated in some grasses, including cereal crops such as maize and rice (Table 1). The maize pollen coat proteome contains 14 unique proteins [17]. In an initial study, a predominant protein of 35 kDa, called *Zm*XYL1, was extracted using diethyl ether and identified by N-terminal sequencing as an acidic endoxylanase [30]. *Zm*XYL1 belongs to glycosyl hydrolase family 10 and displays the highest level of identity (42%) with a 44-kDa barley aleurone xylanase. The tapetum-specific *ZmXYL1* gene transcription begins and peaks at anther developmental stage 2 (young, detached microspores), with levels then sharply decreasing in stage-3 (bicellular pollen grain) and -4 (tricellular pollen grain) anthers. The enzyme is synthesized as a larger 60-kDa precursor and appears and peaks in stage-3 and -4 anthers, respectively, suggesting the existence of a post-transcriptional regulatory mechanism. Proteolysis at the N-terminus by a serine protease yields a mature, active enzyme of 311 AAs [70]. After apoptosis, this enzyme is released with the tapetum remnants to the anther loculus and deposited on the pollen exine surface [70]. An endoxylanase of ~30 kDa was also identified in the pollen coat of Bermuda (*Cynodon dactylon*) and Timothy (*Phleum pratense*) grass [36]. Both proteins share a high level of identity with maize and rice xylanase enzymes and were undetectable in the cytosolic fraction of the cyclohexane-defatted pollen.

Two additional prominent protein bands of 70 and 25 kDa were visible on polyacrylamide gels in diethyl ether-extracted maize pollen coat preparations [30]. The former protein was further identified by micro-sequencing as a novel acidic β-glucanase enzyme [44]. The *ZmGLA3* gene encodes a 644-AA polypeptide of ~69.3 kDa with a putative signal peptide at its N-terminus, suggesting that *Zm*GLA3 protein is secreted from tapetal cells to the anther locule. This maize tapetum-specific β-glucanase differs in its expression pattern from *ZmGLA1* and *ZmGLA2* genes. Thus, *ZmGLA3* transcripts emerged in stage-2 anthers, peaked at stage 3 and then declined at stage 4. ZmGLA3 protein did not appear until stage 3 and peaked in stage-4 anthers. The smaller 25-kDa protein was recently identified as a Cys protease [48]. The tapetum-specific *ZmPCP* gene encodes a single 352-AA polypeptide which, starting from the N-terminus, encloses a 28-AA signal peptide, a peptidase C1A motif-containing 113-AA propeptide and a 211-AA mature protein. *Zm*PCP shares 85% and 58% identity with two closely related protease homologs from sorghum and rice, respectively. *ZmPCP* gene transcription is restricted to the tapetum cells of stage-4 anthers. The *Zm*PCP protein also emerges in stage-4 anthers and later in the pollen coat at stages 5 (mature tricellular pollen grain ready to be released) and M (mature, free pollen grain). These data suggest that *Zm*PCP protease synthesis is regulated at the transcriptional level. Maize Cys protease homologs have recently been identified in the pollen coat eluates of Bermuda, Timothy and Johnson (*Sorghum halepense*) grass [36].

Up to 15 β-expansin genes, which were grouped into two classes, were reported in maize pollen [77]. Class A genes are divided into two complexes (EXPB10 and EXPB11), while class B genes constitute a single complex (EXPB1). Class-B genes encode four basic (pI of 9.1–9.5) glycoproteins of 27–29 kDa in size, named EXPB1, EXPB9, EXPB 10 and EXPB11, which represent distinct forms (a–d) of Zea m 1 allergen [39]. EXPB1 (Zea m 1d) is the most abundant form in the maize pollen grain. Two of these β-expansins, namely EXPB1 (Zea m 1d) and EXPB10 (Zea m 1c), were consistently identified in the maize pollen coat fraction [27,40]. The predicted polypeptides of *EXPB1* and *EXPB10* genes are made up of 269 and 270 amino acids and show approximately 58 and 70% protein sequence identity with ryegrass Lol p 1 allergen, respectively. Expression analysis of both *EXPB1* and *EXPB10* genes showed similar patterns, with low transcript levels at the uninucleate microspore stage and a significant peak after the second mitosis [77,78,79]. The amino acid sequences of EXPB1 and EXPB10 contain a signal peptide at the N-terminus that is split by proteolysis during protein maturation [39]. At the ultrastructural level, Zea m 1d was mainly localized in the tectum and the foot layer of the exine (Table S2), although gold labeling was also occasionally observed in the pollen coat [40]. A novel Cyn d 1 form (β-expansin) was also immunodetected in the pollen coat fraction of the Bermuda grass [36].

In a recent study, Wu *et al.* [27] scaled up maize pollen coat proteome profiling using a chloroform/phenol-based extraction method combined with an MS/MS-coupled two-dimensional electrophoresis (2-DE) strategy. This approach enabled up to 12 unique proteins to be identified (see Table 1). Ten of these were novel maize pollen coat proteins, including: an abscisic acid (ABA)-induced caleosin of 24.3 kDa, a putative 21.6-kDa subtilase, a Rho GDP-dissociation inhibitor 1 of 24.7 kDa, a 23.2-kDa Ras-related protein Rab-2-A and eight allergens recognized as a β-expansin-10 (Zea m 1), a Phl p 2 homologous protein (Zea m 2), three distinct profilins (1–3, Zea m 12) and a cell wall-related exopolygalaturonase enzyme (Zea m 13). Subsequent bioinformatic analysis of the annotated proteins showed that only Zea m 2 was slightly hydrophobic, while the other proteins were moderately hydrophilic, with hydropathicity values ranging from −0.047 to −0.773 [27]. Prediction of subcellular location resulted in the classification of eight of the 12 proteins identified as extracellular. Based on SecretomeP analysis [80], nine proteins were predicted to be secreted. Moreover, as caleosin, Zea m 2 and Cys protease proteins contain transmembrane helices, they may be anchored to the membranous components of the pollen coat.

The diethyl ether-eluted pollen coat fraction of rice was also extensively explored at the proteomic level by [26] using sodium dodecyl sulfate (SDS)-polyacrylamide gel electrophoresis (PAGE) combined with MS/MS analysis. Fourteen reproducible protein bands, with molecular weights ranging from 12 to 70 kDa, were further scrutinized, leading to the identification of 38 unique proteins. As with maize, several β-expansins and expansin-like proteins, a putative tapetum-specific 1,4-β-xylanase of 30 kDa and a ~70-kDa β-glucanase constitute, in that order, the bulk of the rice pollen coat proteome. The pollen coat proteins extracted from *Secale cereale*, *Festuca pratensis* and *Lilium multiflorum* self-sterile and self-fertile plants [81,82], as well as from *Aegilops kotschyi* × *S. cereale* amphiploid plants [83], were also profiled by 2-DE, although these studies lack protein identification data. The proteomic analysis of rapidly released proteins from maize [44], rice [26] and triticale [38] pollen upon hydration led to the identification of novel putative candidates of the pollen coat proteome. Whether these proteins are permanent or transient components of the pollen coat and actively involved in pollination needs to be experimentally verified.

### 2.3. The Pollen Coat Proteome of the Olive Tree

The olive pollen grain is coated with an extracellular lipoid matrix that fills bacular spaces and forms drops on the surface (Figure 1a). This matrix can be stained with the vital stain Nile red to demonstrate its lipidic nature (Figure 1b). We extracted the pollen coat fraction by rinsing fresh pollen grains with cyclohexane (Figure 1c,d) as previously described [29]. Coatless pollen grains were further examined under a transmission electron microscope. They showed no significant ultrastructural alterations in their cell wall or cytoplasm compared with untreated grains (Figure 1c,d). The fluorescein diacetate-based viability test confirmed the integrity of the protoplast and showed similar values compared with the untreated pollen (data not shown). Pollen coat proteins (PCPs) were precipitated in acetone and resuspended in SDS sample buffer [10 mM Tris-HCl (pH 6.8), 1% SDS, 10% glycerol, 40 mM DTT, 0.025% bromophenol blue]. Olive pollen was also placed in a culture solution [0.03% boric acid, 0.01% potassium nitrate, 0.02% magnesium sulfate, 0.03% calcium nitrate, 10% sucrose (pH 5.5–6.5)] and gently rotated for 5 minutes. Then, the medium was filtrated through a 10 μm pore-diameter mesh, and pollen released proteins (PRPs) were processed as above. PCPs and PRPs were further separated using standard SDS-PAGE protocols and labeled with colloidal Coomassie brilliant blue (CBB).

**Figure 2:**
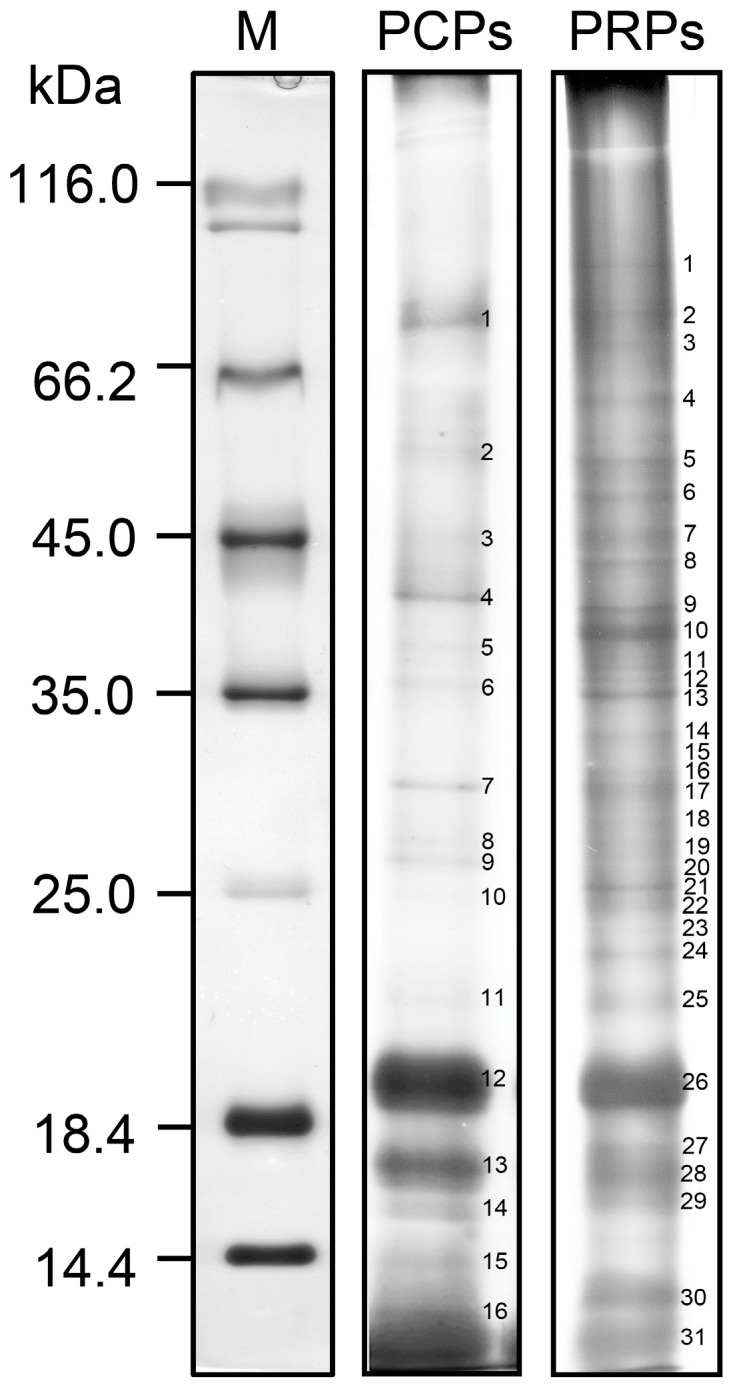
One-dimensional electrophoretic profiles of olive pollen coat proteins (PCPs) and pollen released proteins (PRPs). Protein samples (~20 μg per lane) were separated by SDS-PAGE and stained with CBB. Protein markers (M) are displayed on the left. Numbers in each gel denote the excised bands for MS/MS analysis.

The olive pollen coat proteome consisted of at least 16 PCPs with molecular weights ranging from 10 to 75 kDa, while up to 31 different PRPs were observed in the 8–100 kDa range after analysis of the culture medium (Figure 2). Among the PCPs, two prominent protein bands of ~17 and ~19 kDa, respectively, were by far the most abundant in the olive pollen coat fraction. Two protein bands of similar size were also outlined in the PRP profile. All visible protein bands were manually excised from polyacrylamide gels and subjected to MS/MS analyses following the criteria described previously [84]. Eleven of the 16 PCP bands matched 18 unique proteins (Table S3), including the major olive pollen allergen (Ole e 1) in its different glycosylation states (bands #11–13), several cell wall-degrading enzymes [β-1,3-glucanase (band #2), polygalacturonase (band #3) and two pectinesterases (bands #1,6)], two enzymes of the GDSL serine esterase/lipase family (bands #4,7), a sucrose-degrading invertase (band #1), a protein kinase containing two EF-hand motifs (band #14), a desiccation-related protein (band #6) and a Gly-rich protein homologous to Gly-rich protein 1A (GRP1A) from *Sinapis alba* (band #15). Most of the proteins identified contain a signal peptide in the N-terminus and are predicted to have an apoplastic or extracellular location (Table S1). Most of the PCPs were also identified in the culture medium fraction, supporting the idea that they are released from the pollen surface upon its contact with the aqueous medium. As expected, several Ole e 1 isoforms were identified among the PCPs, showing point substitutions in their amino acid sequences [85,86,87]. In the case of PRPs, we positively identified 169 unique proteins (Table S3). We observed a high level of polymorphism, with up to 36 protein families being represented by at least two or more different protein forms. Approximately 15% and 19% of the proteins identified were predicted to locate in the apoplast/extracellular space and to contain a signal peptide, respectively.

## 3. Molecular and Biological Functions of the Pollen Coat-Associated Proteins

### 3.1. Pollen Adhesion and Hydration

Upon their arrival at a receptive stigmatic surface, pollen grains adhere within a period of seconds. Cell–cell adhesion is one of the key factors influencing effective pollen performance. In dry stigmas, pollen adhesion, which is a feature of the pollen grain, is highly selective [88]. Thus, stigmas of the *Brassicaceae* adhere poorly to foreign pollen grains [89]. In *Arabidopsis* and *Brassica* species, the nature of pollen-stigma adhesions changes becoming stronger as pollination progresses [89,90,91]. Early pollen-stigma adhesion is very rapid and involves lipophilic interactions between the stigma surface and the pollen exine [89]. Within a few minutes, the pollen coat is mobilized from the exine baculae to form a foot-like structure at the contact site that enhances pollen-stigma adhesion through protein-protein interactions. Thus, PCP-A1, a pollen coating-borne peptide, was found to bind to the stigma wall-anchored SLG protein in *B. oleracea* [29,59]. In *Brassica campestris*, SLG-like 1 (SLR1), a stigma-specific secreted glycoprotein not linked to the S-locus, binds with high affinity to two almost identical members of the class A pollen coat proteins (PCP-A), called SLR1-BP1 and SLR1-BP2 [20]. In *Brassica napus*, an amphidiploid hybrid of *B. oleracea* and *B. campestris*, a 7-kDa PCP-A1-like protein was also shown to interact with SLR1 [58]. In plants with a wet stigma, pollen capture and adhesion is likely to be mediated mainly by the stickiness and surface tension of the stigma exudate [92].

After binding to the stigmatic surface, desiccated pollen grains need to hydrate in order to germinate a pollen tube. In dry-type stigmas, the onset of pollen hydration is assisted by the lipid-rich pollen coat, which is mobilized to form a contact zone (*i.e.* the foot) at the pollen-stigma boundary [93]. Thus, *Arabidopsis* plants with impaired pollen hydration, such as cer mutants, are defective in terms of the lipid content level and composition of their pollen coat [21,89,94,95]. A pioneer study has also suggested that pollen coat proteins play an important role in pollen hydration [22]. *Arabidopsis* glycine-rich protein 17 (GRP17) is a tapetum-specific oleosin (T-oleosin) that contains 21% of the total coat protein. The pollen of *grp17-1* mutants lacks detectable levels of GRP17 protein on the coating, which is similar in appearance and lipid composition to that of wild-type pollen. The *grp17-1* mutants also appear to be normal in terms of pollen production, percentage of viable pollen, pollen adhesion and pollen tube performance, number of seeds per silique and seed viability values. However, the onset of pollen hydration and subsequent steps are significantly delayed in *grp17-1* pollen. This delay in hydration is probably due to the failure of pollen to interact with the stigma rather than to its inability to absorb water. Since pollination takes less than one hour, this short delay impairs the ability of *grp17-1* pollen to compete with wild-type pollen [22]. Pollen coat-associated oleosins, which are absent in the pollen coat of other species with a dry stigma such as maize and rice, seem to be a distinctive feature of the *Brassicaceae* family [26,44].

Two recent studies have demonstrated that T-oleosins are essential for tapetosome formation in *Brassicaceae* [14,66]. Huang *et al.* [14] reported that *Arabidopsis* mutants of T1 and T3−T6 oleosin paralogs showed a consistent loss of organized tapetosome structures and pollen coat materials such as flavonoids as well as an impaired ability on the part of pollen to tolerate dehydration and to germinate *in vitro*. Complementation analysis of the *∆T3* mutant restored the wild-type phenotype, thus confirming that the anomalous phenotype resulted from loss of T3 oleosin function. Similarly, T5 NF-green fluorescent protein (GFP) translational fusion also resulted in smaller, partially developed tapetosomes and lower levels of T5 in the pollen coat [66]. It was concluded that oleosin targeting is affected in transformed plants probably because GFP cannot fully mimic the C-terminal mature domain. *Cleome hassleriana*, a closely-related *Brassicaceae* species of the *Cleomaceae* family, lacks the T-oleosin cluster, identifiable tapetosomes and dehydration-tolerant pollen. Interestingly, transformation of *C. hassleriana* using the *Arabidopsis* T3 oleosin gene resulted in the assembly of primitive tapetosomes, which consisted of lipid droplets and flavonoid-containing vesicles [14]. Transformed plants also contained pollen tolerant to desiccation. All these findings suggest that oleosin redundant paralogs created tapetosomes and conferred adaptive quantitative benefit of pollen vigor in *Brassicaeae* [14].

Two extracellular lipases, called EXL4 and EXL6, were also identified in the pollen coat of *A. thaliana* [24]. The function of one of these enzymes, namely EXL4, has recently been examined [49]. The pollen grain of *exl4-1* mutants lacks EXL4 lipase on the coating, which has a comparable structure and lipid composition to that of the wild-type pollen. The *exl4-1* plants also appear to be normal, and pollen performance is not compromised. Wild-type pollen begins hydration very rapidly upon contact with the stigmatic surface, while *exl4-1* pollen barely delays the initiation of water uptake. However, this delay is more pronounced when the hydration process is completed. Moreover, *exl4-1* pollen is at a disadvantage when challenged by *EXL4-1* pollen on the stigma. This phenotype differs from that of *grp17-1* pollen, which delays the onset of hydration but shows no defects with respect to completing the hydration process relative to wild-type pollen. The *exl4-1/grp17-1* double mutants showed an additive defect at the initiation of hydration although its completion was normal [49]. This suggests that GRP17 and EXL4 may act synergistically to modify lipid composition at the pollen-stigma interface. It has been hypothesized that the GRP17 oleosin domain may initially change the biophysical properties of lipids, thus increasing their solubility. EXL4 and other pollen coat lipases further hydrolyze emulsified lipids, which changes the permeability of the cuticle and the pollen coating, thus facilitating the capillary diffusion of water from the stigma to the desiccated pollen [96].

On wet stigmas, pollen grains land on an extracellular secretion called the stigmatic exudate [84,97]. In some species such as the olive tree, this secretion facilitates pollen hydration without direct contact with the papillary cells [97]. In tobacco, however, the pollen grain sinks through the exudate and establishes direct contact with the papillary cell wall and/or other grains [98]. This suggests that water may be transported from the papillae to the pollen grains, as well as from grain to grain. The ablation of the stigma’s secretory tissue impairs pollen hydration and leads to female sterility in tobacco, although the exogenous application of the exudate restores fertility [19,99]. These data suggest that the stigmatic exudate is functionally analogous to the pollen coat. The addition of *cis*-unsaturated triacylglycerides is sufficient to promote pollen hydration, thus suggesting that lipids regulate the flow of water from the pistil to the pollen grain [19,98]. Unlike dry-type species, plant species with wet stigmas lack pollen coat oleosins [28,100,101]. These data suggest that, on wet stigmas, pollen hydration may be modulated by another set of proteins. Alternatively, this process may not be subject to regulation, which closely correlates with the fact that wet-type stigmas are more permissive with respect to hydration of incompatible pollen [96].

### 3.2. Pollen–Stigma Communication

Pollen hydration is highly-regulated in the *Brassicaceae* species and provides the plant with a subtle mechanism to recognize and reject incompatible pollen [93,96]. In the *Brassicaceae* family, the pollen-S determinant is a small basic Cys-rich protein, called SP11/SCR, which is synthesized and secreted from the tapetum and deposited on the pollen surface as a constituent of the pollen coat [64]. SP11/SCR interacts with the stigma S determinant located at the plasma membrane of papillary cells [102]. S-haplotype-specific binding of SP11/SCR to SRK triggers a self-incompatibility response and the rejection of self-pollen by blocking its hydration and germination [103,104].

**Figure 3:**
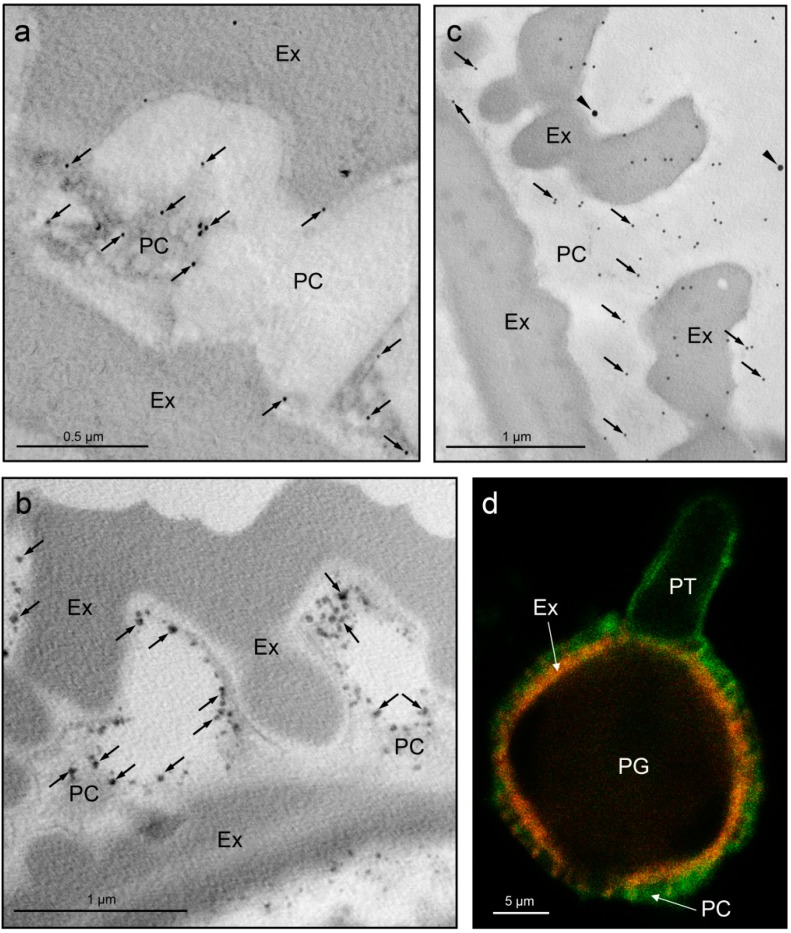
(**a**) Caleosin immunostaining (arrows) in the olive mature pollen; (**b**) Acetyl cholinesterase activity associated with the pollen coat material in olive mature pollen; (**c**) Double immunolabeling of Ole e 1 (10-nm gold particles, arrows) and profilin (30-nm gold particles, arrowheads) allergenic proteins in the olive pollen coat; (**d**) Fluorescent immunostaining of arabinogalactan proteins (AGPs) in germinated olive pollen using an antibody against the JIM13 epitope. Green fluorescence appears at the pollen surface, associated with the pollen coat, and the pollen tube wall. Red signal corresponds to pollen wall autofluorescence. Abbreviations: Ap, aperture; Ex, exine; PC, pollen coat; PG, pollen grain; PT, pollen tube. Figure 3a reproduced with some modifications from [15].

Caleosins were first reported in the pollen coat of *Brassicaceae* [24,25]. Moreover, a 30-kDa caleosin, which is synthesized in the anther tissues, has recently been characterized in olive pollen [15]. The protein was first localized in the tapetal cells at the free microspore stage. As anthers developed, tapetal cells showed the presence of structures composed of caleosin-containing lipid droplets closely packed and enclosed by ER-derived cisternae and vesicles (Table S2). After tapetal cells lost their integrity, the caleosin-containing remnants of the tapetum were released to the loculus and filled the cavities of the mature pollen exine to form the pollen coat (Figure 3a) [15]. Caleosins possess a calcium-binding domain consisting of a conserved EF-hand capable of binding a single calcium atom, a central hydrophobic region with a potential lipid-binding domain and a C-terminal region containing several putative protein kinase phosphorylation sites [105]. The presence of these signaling-related motifs and its extracellular location suggest that the pollen coat-associated caleosin may play a role in pollen–stigma communication during pollination.

The pollen coat of broad bean and olive displays high levels of acetyl cholinesterase (AChE; EC 3.1.1.7) activity as revealed by cytochemical techniques (Figure 3b, Table S2) [32,33]. Although plant AChEs differ with respect to their sequences from those of animals, they conserve the catalytic triad and regulate the level of acetylcholine (ACh) in a manner similar to that of the animal enzyme [106]. Tezuka *et al.* [107] found that the exogenous supply of ACh promoted *in vivo* pollen tube growth in *Lilium longiflorum* after self-incompatible pollination. Neostigmine, a potent inhibitor of AChE, also exerts the same effect. Thus, self-pollinated pistil and self-pollen showed higher AChE activity levels than cross-pollinated pistil and cross pollen [107]. Given these findings, the authors of this study hypothesized that self-incompatibility in Eastern lily occurs as a result of a decrease in ACh concentrations, whose level is regulated by AChE. Although it is tempting to suggest that a similar mechanism might exist in broad bean and olive, to date, no experimental evidence has confirmed its existence.

Other pollen coat proteins are also putative candidates to participate in pollen-stigma signaling processes. Polygalacturonase-mediated hydrolysis of homogalacturonans yields oligogalacturonides, which can act as signaling molecules [108]. As some pollen coat lipases perform acyl transfer reactions in extracellular environments [109], they might participate in certain yet-to-be-discovered signaling-related activities.

### 3.3. Pollen Germination

Following pollen hydration, a pollen tube emerges from the aperture and elongates through its apex within the stigma before entering the style. On dry stigmas, the stigmatic surface is typically enclosed by a continuous lipidic cuticle that is breached by a particular group of pollen esterases, called cutinases, which are secreted to the extracellular space [110]. After breaking this barrier, the pollen tube penetrates the papilla cell wall aided by the action of hydrolytic enzymes that degrade pectins and other cell wall polysaccharides. In wet-type stigmas, the pollen tube grows through the exudate-filled intercellular spaces. Remarkably, the stigmatic secretion is also rich in cell wall-loosening enzymes [84].

Β-expansins and expansin-like allergens have been identified in the pollen coat of several grasses including maize, rice and Bermuda grass (Table 1). The function of Zea m 1d (EXPB1), the most abundant Zea m 1 form in the maize pollen coat, has recently been studied [42,111]. The *expb1/expb1* mutant shows a reduction of ~30% in the EXPB1 pool but has no apparent effect on pollen viability, and pollen tube performance is not compromised [112]. Pollen tubes from *expb1/expb1* and EXPB1/EXPB1 plants elongate at similar rates *in vitro* although the pollen from heterozygous plants grows faster probably due to the heterosis effect. Thus, *expb1* pollen, whose capacity to penetrate the silk is reduced, grows more slowly than EXPB1 pollen in the style. Such differences in vigor impair the ability of *expb1* pollen to compete with wild-type pollen on the stigma and explain why its reproduction is less successful [42,111]. In addition, *expb1* pollen forms aggregates inside the anther, which prevents proper pollen dispersal [42]. At the molecular level, maize pollen-specific β-expansins, though not *bona fide* lytic enzymes, act as cell wall-loosening agents [41]. The effect of pollen β-expansins on silk cells results in the solubilization of cell wall matrix polysaccharides in a selective and non-enzymatic manner [43]. Β-expansins also induce the stress relaxation and irreversible extension of the primary wall, thus promoting pollen tube entry through the stigma and style tissues, most likely by weakening the middle lamella. A 10-kDa expansin-like protein (Zea m 2) homologous to the Phl p 2 precursor protein has also recently been identified in the pollen coat fraction of maize [27]. This protein contains a cellulose binding-like domain (CBD), but not the distinctive catalytic domains of an expansin, and is released from pollen to the extracellular space during germination [44]. The exogenous addition of the CBD at low concentrations enhances peach pollen tube elongation *in vitro*, which is likely to delay cellulose crystallization at the pollen tube tip [112].

Some cell wall-degrading enzymes, including xylanases and β-glucanases, are also associated with the pollen coat (Table 1). The occurrence of tapetum-specific xylanases in maize and rice, though not in other plants, is probably connected with the unique polymer composition of grass cell walls [113]. To study the function of pollen coat xylanases, Suen and Huang [71] generated a transgenic maize line, called xyl-less, lacking ZmXYN1, on the pollen coat. Xyl-less plants were not phenotypically distinct from wild-type plants in terms of pollen coat morphology or lipid composition, while mutant pollen tubes were as competent as wild-type tubes in germinating and growing *in vitro*. However, as the solidity of the germination medium increased, xyl-less pollen germinated less efficiently than wild-type pollen after 20 min [71]. Such differences were even more striking with respect to silk, reaching a 5/1 ratio, although the disparity decreased over longer germination periods before disappearing altogether. Thus, in maize, the removal of the pollen coat xylanase impaired the onset of germination. The ability of xyl-less pollen to breach the silk surface was also reduced as compared with wild-type pollen, and the pollen tube showed an atypical curved growth pattern [71]. Interestingly, when the silk surface was supplied with exogenous xylanase, the efficiency of xyl-less pollen tubes to enter the silk returned to levels similar to those of the wild-type pollen tubes. At the molecular level, maize pollen coat xylanase is an endohydrolase, with the highest activity levels being observed at pH 5.0 on oat-spelled xylan [30]. During anther development, xylanase activity closely correlates with the expression pattern of the 35-kDa active enzyme but not with the inactive 60-kDa precursor [70]. ZmXYN1 activity appears at the vacuolated microspore stage, peaks when pollen reaches maturity and decreases to half in germinated pollen. Xylose inhibits pollen coat xylanase activity and hinders pollen tube penetration on the stigma [71]. Pollen coat xylanases therefore help the pollen tube to breach the silk by degrading cell wall xylans, thus simultaneously providing new cell wall precursors of the tube.

Β-glucanases are important components of the pollen coat proteome (Table 1). Β-glucanases are involved in the cleavage of glucan polymers to glucooligosaccharides and glucose units. In maize, a tapetum-derived 1,3-β-glucanase, called *Zm*GLA3, is highly active at a pH range of 5.0-5.5 on various substrates including laminarin and lichenan [44]. This pollen coat-associated β-glucanase gene is distinct from that involved in the hydrolysis of the callose wall that encloses microspores in the tetrad. Likewise, Ole e 9 allergen is an olive pollen-specific 1,3-β-endoglucanase with optimal activity at a similar pH range [114]. These enzymes may not only be involved in degrading the tapetum cell wall but also in breaking the stigma cell wall for the entry of the pollen tube given that these enzymes are released into the extracellular space after pollen germination (Table S1). Interestingly, as the exogenous application of β-glucanases increases pollen tube growth rates *in vitro* [115], other functions cannot be ruled out. For instance, β-glucanases might modulate callose degradation just behind the tube tip to prevent its blockage and to facilitate its expansion [116]. Alternatively, β-glucanases may amend the porosity of the callosic layer, thus regulating the molecular exchange between the pollen tube and the stigmatic cells.

A number of pectin-degrading enzymes, including pectin methylesterases, polygalacturonases and pectate lyases, were also identified as putative elements of the pollen coat and thecal orbicules (Table 1). These hydrolytic enzymes are released into the extracellular medium following pollen hydration [44,55]. Consequently, they may degrade pectins, thus assisting the pollen tube to penetrate the papillar cell wall upon compatible pollination [25,57]. However, other functions, such as promoting pollen tube wall extension or providing the pollen tube with new wall precursors, should not be ruled out [115]. In dry stigmas, in addition to cutinases, other esterolytic enzymes such as pollen coat lipases may also be involved in breaking down the lipidic constituents of the cuticle in order to facilitate pollen tube penetration.

### 3.4. Expanding the List of Pollen Coat Proteins and Functions

The pollen coat carries certain proteins with, as yet, no assigned function (Table 1). For instance, the main protein constituent of the olive pollen coat is the allergen Ole e 1 (Figure 2, bands #11–13), as shown by immunostaining (Table S2) and proteomic experiments (Figure 3c and Table S3). Although Ole e 1 may account for up to 20% of the pollen total protein [117], its molecular and biological function remains elusive. Ole e 1 shares some features with the LAT52 protein including ~36% sequence identity, a similar size, their glycosylated residues and secretory nature, thus supporting the idea that they may be functionally homologous [118]. LAT52 interacts with two pollen receptor kinases (PRK), *Le*PRK1 and *Le*PRK2, located in the plasma membrane of the tomato pollen tube [119]. Following pollen germination in the stigma, LAT52 is displaced by a pistil protein called STIL, which triggers an autocrine signaling cascade in the pollen tube [120], thus regulating its protrusion and apical growth through the pistil tissues in tomato.

Cysteine proteases seem to be common constituents of the pollen coat in grasses (Table 1 and Table S2). Since these proteins are expressed at a late stage in anther development, it has been suggested that they may be related to programmed cell death (PCD) of the tapetum, either alone or coordinately with other proteases such as subtilisin-like Ser proteases [121]. A Cys protease from *Arabidopsis*, called CEP1, has recently been shown to be an executor directly involved in tapetal apoptosis [47]. Thus, CEP1 function loss blocked cell wall disintegration of tapetal cells and reduced fertility due to abnormal pollen exine deposition. The subcellular location of Cys proteases in vacuoles (Table S2) is also closely in line with the current model of vacuole-mediated apoptosis in plant cells [47,48]. Although pollen-coat Cys proteases may be non-functional leftovers of the tapetum, they may play an active role in the stigma by participating in controlled proteolysis events important for pollen adhesion and recognition. Alternatively, these proteases might generate or modify compounds such as peptides that act as pollen tube guidance signals.

Two proteins identified in the pollen coat of *A. thaliana* proved to be similar to receptor-like protein kinases. However, as these proteins lack a kinase domain, their role in signaling events is unlikely. On the other hand, these proteins contain salt stress/antifungal (Pfam: PF01657) motifs and therefore might be involved in antifungal activity on the pollen surface. Intense immunostaining with the JIM13 antibody was also observed in the olive pollen coat material, suggesting the presence of arabinogalactan proteins (AGPs) in this coating (Figure 3d) [35]. These extracellular glycoproteins are probably synthesized in the anther tapetum [122]. Interestingly, AGPs have been described as key components of the stigmatic exudate and stylar extracellular secretion [84,123], with respect to which they have been postulated as candidate molecules for pollen-pistil adhesion phenomena. Calcium-binding proteins similar to calmodulins have also been identified in the pollen coat of *B. rapa* [46] and olive (Table S1). Despite the puzzling nature of the function of these intracellular proteins, it has been speculated that they might be released at the contact zone and cause a change in pollen grain calcium distribution, which is likely to trigger germination [46]. It is not clear whether other putative pollen-coat proteins, such as profilins and Phl p 4, play a role in pollination.

## 4. Conclusions

The pollen coat, the outermost layer of the pollen grain, protects the male gametophyte from the environment and plays a key role in pollination. To date, only a small number of proteins have been definitively identified as inherent to this extracellular coating and functionally characterized in greater detail. Pollen coat proteins are essential for pollen adhesion and hydration, pollen–stigma recognition and communication, pollen germination and stigma invasion. The pollen coat proteome has three main features. Firstly, its composition appears to be consistent between closely-related species in a family but differs considerably between divergent botanical families. These discrepancies probably reflect functional divergence and could be mainly explained by the stigma’s diverse structural features (dry *vs* wet-type, for example). Secondly, some pollen-coat proteins show considerable multiplicity of forms that are presumed to have different biochemical functions in terms of substrates or contribute to the specificity of the pollen–stigma recognition process. This wide range of forms is due to multiple genes and alleles or alternative splicing during transcription, post-translational modifications, such as glycosylation, and protein processing. In addition, the presence of multiple forms in different species within a family points to the maintenance of this trait due to selection pressure. Finally, many of these protein constituents are capable of evoking an IgE-mediated immune response in humans, thus highlighting the importance of the pollen coat as also a source of aeroallergens. This should be taken into account for commercial extract standardization, which is the basis of current diagnosis and vaccination procedures. Further studies in the future of other species and families would probably expand the list of pollen-coat proteins and functions.

## Supplementary Materials

The supplementary materials are available online at www.mdpi.com/2227-7382/4/1/5/s1. Table S1: Species discussed in Table 1 and type of stigma. Note that most of the species studied possess a dry-type stigma; Table S2: Subcellular localization of proteins reviewed in Table 1 on the basis of the enzyme and immunocytochemical data; Table S3a: Olive pollen coat proteins identified by nano LC-MS/MS sequencing; Table S3b: Olive pollen proteins released to the extracellular medium identified by nano LC-MS/MS sequencing.
