# Supplementary material for: The Pollen Coat Proteome: At the Cutting Edge of Plant Reproduction
Source: Proteomes. 2016 Jan 29;4(1):5. doi: 10.3390/proteomes4010005 (PMC5217362; doi:10.3390/proteomes4010005)
Supplement: Supplementary file 1 [file proteomes-04-00005-s001.zip › proteomes-113892-supplementary_Tables S1 and S2_Final.pdf]

Supplementary Materials: The Pollen Coat Proteome: At the Cutting Edge of Plant Reproduction

Juan David Rejón, François Delalande, Christine Schaeffer-Reiss, Juan de Dios Alché, María Isabel Rodríguez-García, Alain Van Dorsselaer and Antonio Jesús Castro

Table S1. Species discussed in Table 1 and type of stigma. Note that most of the species studied possess a dry-type stigma.

Species	Type of Stigma
Arabidopsis thaliana	Dry
Brassica carinata	Dry
Brassica napus	Dry
Brassica oleracea	Dry
Brassica rapa	Dry
Crytomeria japonica	NA
Cupressus arizonica	NA
Cupressus sempervirens	NA
Cynodon dactylon	Dry
Helianthus annuus	Wet
Olea europaea	Wet
Nicotiana tabacum	Wet
Oryza sativa	Dry
Phleum pretense	Dry
Sorghum halepense	Dry
x*Triticosecale*	Dry
Vicia faba	Dry
Zea Mays	Dry

NA, not applicable.

Table S2. Subcellular localization of proteins reviewed in Table 1 on the basis of the enzyme and immunocytochemical data.

Protein Name	Species (Method [1])	Subcellular Location [2]	References
**AChE**			
Acetylcholinesterase	*Olea europaea* (EC)	Ap/Cy/PC/PTC/PTW	[1]
Cholinesterase	*Vicia faba* (EC)	Ap/PC/PTC/PTW	[2]
**AGP-JIM13**	*O. europaea* (CM/TEM)	Ap/Cy/GCW/PC/PTC/PTW	[3]
**Beta-expansins**			
Phl p 1 allergen	*Phleum pretense* (TEM)	Cy/Ex/Nu/PC	[4]
Zea m 1 allergen	*Zea mays* (TEM)	Cy/Ex/PC	[5]
**Caleosin**	*O. europaea* (CM/TEM)	Ex/LB/PC/PTC/PTM/PTV/TER/TLB	[6,7]
**Calmodulin-like**			
Bra r 1	*Brassica rapa* (LM/TEM)	Cy/PC/PS/TC	[8]
**Cys proteases**			
CEP1	*Arabidopsis thaliana* (TEM)	TCW/Ex/PC/TC/TV	[9]
Cysteine protease	*Zea Mays* (CM)	PS/TV	[10]
**Ole e 1**	*Olea europaea* (FM/TEM)	Ap/ER/Ex/PC/PTC/TER	[11,12]
**Pectate lyases**			
Cry j 1	*Cryptomeria japonica* (TEM)	GCW/Ex/Go/PC/TR	[13]
Cup a 1	*Cupressus arizonica* (TEM)	Cy/Ex/Go/In/LB/Nu/Or/PC/V	[14]
Cry j 1-like	*C. sempervirens* (TEM)	Cy/Ex/Go/In/LB/Nu/Or/PC/V	[14]
**Phl p 4**	*Phleum pretense* (TEM)	PC/Cy	[15]
**Polygalaturonase**	*Brassica napus* (LM)	PS/PTC	[16]

**SP11/SCR**	*Brassica rapa* (SEM/TEM)	Cy/Ex/PC/TC	[17,18]
**Profilins**			
Ole e 2	*Olea europaea* (TEM)	Cy/Ex/PC	[19]
**T-oleosins**			
BnOlnB;4	*Arabidopsis thaliana* (TEM)	PC/Ta/TER/TLB	[20]
BnOlnB;4	*Brassica carinata* (TEM)	PC/Ta	[21]
BnOlnB;3–4	*Brassica napus* (TEM)	PC/TLB	[22]
BnOlnB;3–4	*Brassica napus* (CM)	PS/Ta/TER/TLB	[23]

[1] CM, confocal microscopy; EC, enzyme cytochemistry; FM, fluorescence microscopy; LM, light microscopy; SEM, scanning electron microscopy; TEM, transmission electron microscopy; [2] AP, pollen aperture; Cy, pollen vegetative cell cytoplasm; ER, pollen endoplasmic reticulum; Ex, pollen exine; GCW, pollen generative cell wall; Go, pollen Golgi apparatus; In, pollen intine; LB, pollen lipid body; Nu, vegetative cell nucleus; Or, pollen orbicule; PC, pollen coat; PS, pollen surface; PTC, pollen tube cytoplasm; PTM, pollen tube plasma membrane; PTV; pollen tube vacuole; PTW, pollen tube wall; Ta, tapetosome; TC, tapetum cytoplasm; TCW, tetrad callose wall; TER, tapetum ER; TLB, tapetum lipid body; TR, thecal remnants; TV, tapetum vacuole; V, vegetative cell vacuole.

References

1. Rejón, J.D.; Zienkiewicz, A.; Rodríguez-García, M.I.; Castro, A.J. Profiling and functional classification of esterases in olive (*Olea europaea*) pollen during germination. *Ann. Bot.* **2012**, *110*, 1035–1045.
2. Bednarska, E. The localization of nonspecific esterase and cholinesterase activity in germinating pollen and in pollen tube of *Vicia faba*. I. The effect of actinomycin-D and cycloheximide. *Biol. Plant.* **1992**, *34*, 229–240.
3. Castro, A.J.; Suárez, C.; Zienkiewicz, K.; Alché, J.D.; Zienkiewicz, A.; Rodríguez-García, M.I. Electrophoretic profiling and immunocytochemical detection of pectins and arabinogalactan proteins in olive pollen during germination and pollen tube growth. *Ann. Bot.* **2013**, *112*, 503–513.
4. Staff, I.A.; Taylor, P.E.; Smith, P.; Singh, M.B.; Knox, R.B. Cellular localization of water-soluble, allergenic proteins in rye-grass (*Lolium perenne*) pollen using monoclonal and specific IgE antibodies with immunogold probes. *Histochem. J.* **1990**, *22*, 276–290.
5. Wang, W.; Milanesi, C.; Faleri, C.; Cresti, M. Localization of group-1 allergen Zea m 1 in the coat and wall of maize pollen. *Acta Histochem.* **2006**, *108*, 395–400.
6. Zienkiewicz, K.; Zienkiewicz, A.; Rodríguez-García, M.I.; Castro, A.J. Characterization of a caleosin expressed during olive (*Olea europaea* L.) pollen ontogeny. *BMC Plant Biol.* **2011**, *11*, 122.
7. Zienkiewicz, K.; Castro, A.J.; Alché, J.D.; Zienkiewicz, A.; Suarez, C.; Rodríguez-García, M.I. Identification and localization of a caleosin in olive (*Olea europaea* L.) pollen during *in vitro* germination. *J. Exp. Bot.* **2010**, *61*, 1537–1546.
8. Okada, T.; Zhang, Z.J.; Russell, S.D.; Toriyama, K. Localization of the Ca^{2+}-binding protein, Bra r 1, in anthers and pollen tubes. *Plant Cell Physiol.* **1999**, *40*, 1243–1252.
9. Zhang, D.; Liu, D.; Lv, X.; Wang, Y.; Xun, Z.; Liu, Z.; Li, F.; Lu, H. The cysteine protease CEP1, a key executor involved in tapetal programmed cell death, regulates pollen development in *Arabidopsis*. *Plant Cell* **2014**, *26*, 2939–2961.
10. Li, Y.; Suen, D.F.; Huang, C.Y.; Kung, S.Y.; Huang, A.H.C. The maize tapetum employs diverse mechanisms to synthesize and store proteins and flavonoids and transfer them to the pollen surface. *Plant Physiol.* **2012**, *158*, 1548–1561.
11. Alché, J.D.; Castro, A.J.; Olmedilla, A.; Fernández, M.C.; Rodríguez, R.; Villalba, M.; Rodríguez-García, M.I. The major olive pollen allergen (Ole e I) shows both gametophytic and sporophytic expression during anther development, and its synthesis and storage takes place in the RER. *J. Cell Sci.* **1999**, *112*, 2501–2509.
12. Alché, J.D.; M´rani-Alaoui, M.; Castro, A.J.; Rodríguez-García, M.I. Ole e 1, the major allergen from olive (*Olea europaea* L.) pollen, increases its expression and is released to the culture medium during *in vitro* germination. *Plant Cell Physiol.* **2004**, *45*, 1149–1157.
13. Miki-Hirosige, H.; Nakamura, S.; Yasueda, H.; Shida, T.; Takahashi, Y. Immunocytochemical localization of the allergenic proteins in the pollen of *Cryptomeria japonica*. *Sex. Plant Reprod.* **1994**, *7*, 95–100.

14. Suárez-Cervera, M.; Takahashi, Y.; Vega-Maray, A.; Seoane-Camba, J.A. Immunocytochemical localization of Cry j 1, the major allergen of *Cryptomeria japonica* (*Taxodiaceae*) in *Cupressus arizonica* and *Cupressus sempervirens* (*Cupressaceae*) pollen grains. *Sex. Plant Reprod.* **2003**, *16*, 9–15.
15. Fischer, S.; Grote, M.; Fahlbusch, B.; Muller, W.D.; Kraft, D.; Valenta, R. Characterization of Phl p 4, a major timothy grass (*Phleum pratense*) pollen allergen. *J. Allergy Clin. Immunol.* **1996**, *98*, 189–198.
16. Dearnaley, J.D.W.; Daggard, G.A. Expression of a polygalacturonase enzyme in germinating pollen of *Brassica napus*. *Sex. Plant Reprod.* **2001**, *13*, 265–271.
17. Shiba, H.; Takayama, S.; Iwano, M.; Shimosato, H.; Funato, M.; Nakagawa, T.; Che, F.S.; Suzuki, G.; Watanabe, M.; Hinata, K.; Isogai, A. A pollen coat protein, SP11/SCR, determines the pollen S-specificity in the self-incompatibility of *Brassica* species. *Plant Physiol.* **2001**, *125*, 2095–2103.
18. Iwano, M.; Shiba, H.; Funato, M.; Shimosato, H.; Takayama, S.; Isogai, A. Immunohistochemical studies on translocation of pollen S-haplotype determinant in self-incompatibility of *Brassica rapa*. *Plant Cell Physiol.* **2003**, *44*, 428–436.
19. Morales, S.; Jiménez-López, J.C.; Castro, A.J.; Rodríguez-García, M.I.; Alché, J.D. Olive pollen profilin (Ole e 2 allergen) co-localizes with highly active areas of the actin cytoskeleton and is released to the culture medium during *in vitro* pollen germination. *J. Microsc.* **2008**, *231*, 332–341.
20. Lévesque-Lemay, M.; Chabot, D.; Hubbard, K.; Chan, J.K.; Miller, S.; Robert L.S. Tapetal oleosins play an essential role in tapetosome formation and protein relocation to the pollen coat. *New Phytol.* **2016**, *209*, 691–704.
21. Foster, E.; Schneiderman, D.; Cloutier, M.; Gleddie, S.; Robert, L.S. Modifying the pollen coat protein composition in *Brassica*. *Plant J.* **2002**, *31*, 477–486.
22. Murphy, D.J.; Ross, J.H.E. Biosynthesis, targeting and processing of oleosin-like proteins, which are major pollen coat components in Brassica napus. *Plant J.* **1998**, *13*, 1–16.
23. Hsieh, K.; Huang, A.H.C. Lipid-rich tapetosomes in Brassica tapetum are composed of oleosin-coated oil droplets and vesicles, both assembled in and then detached from the endoplasmic reticulum. *Plant J.* **2005**, *43*, 889–899.

© 2016 by the authors; licensee MDPI, Basel, Switzerland. This article is an open access article distributed under the terms and conditions of the Creative Commons by Attribution (CC-BY) license (http://creativecommons.org/licenses/by/4.0/).
